# Pineal gland seminoma: unusual localisation investigated through a case report and a literature review

**DOI:** 10.3332/ecancer.2025.1918

**Published:** 2025-05-29

**Authors:** F Z Abdelli, S Ouaddane Alami, S Khalfi, K Soussy, W Hassani, F Z Farhane, Z Alami, T Bouhafa

**Affiliations:** Radiation Therapy Department, Hassan II University Hospital, Fes 30050, Morocco

**Keywords:** chemotherapy, pineal gland, radiotherapy, seminoma

## Abstract

Germinoma (called seminoma in the testis and dysgerminoma in the ovary) is the neoplastic side of the primitive germ cell. It may be of gonadal or extragonadal origin, although intracranial location remains rare (between 0.5% and 3.2% in adults), 2/3 of which are located in the pineal region. We report a case of a patient with a seminoma of the pineal gland. Symptoms included headache, vomiting and diplopia. The work-up was negative. The patient was initially treated with bleomycin, etoposide and platinum protocol chemotherapy. The tumour was deemed inaccessible to surgery, which is why the patient underwent three-dimensional conformal radiotherapy with a dose of 24 Gy in 12 fractions to the tri-ventricular system, followed by a 16 Gy boost to the pineal region. Pineal neoplasms are rare. Its prognosis has been considerably improved thanks to a multidisciplinary approach. However, radiotherapy remains a good alternative approach.

## Background/introduction

Germ cell tumours (GCTs) of the pineal region are rare. They account for 0.5% to 1% of adult intracranial tumours [1]. Germinoma accounts for 50% [2]. These tumours develop from primitive germ cells (PGCs), of which they constitute the neoplastic side.

The deep cerebral location and the variety of histological subtypes explain the interest in these lesions and the difficulties in treating them. Their management is multidisciplinary, involving surgery, radiosurgery, radiotherapy and chemotherapy [1].

We report here on a rare case of seminoma of the pineal gland in a young adult, non-surgical treated using chemotherapy plus radiotherapy in which we outline the main clinical and radiological features and the various treatment options, supported by a review of the literature.

## Clinical case

Our 17-year-old patient had no notable medical history and had never undergone surgery prior to the current treatment. There was no family history of tumours or neurological disorders, suggesting a generally preserved state of health. He initially presented with symptoms of intracranial hypertension, including headaches associated with progressive vomiting. After 2 months, his condition worsened with the onset of diplopia-type visual disturbances, although an ophthalmological examination revealed no abnormalities.

**Figure 1.**
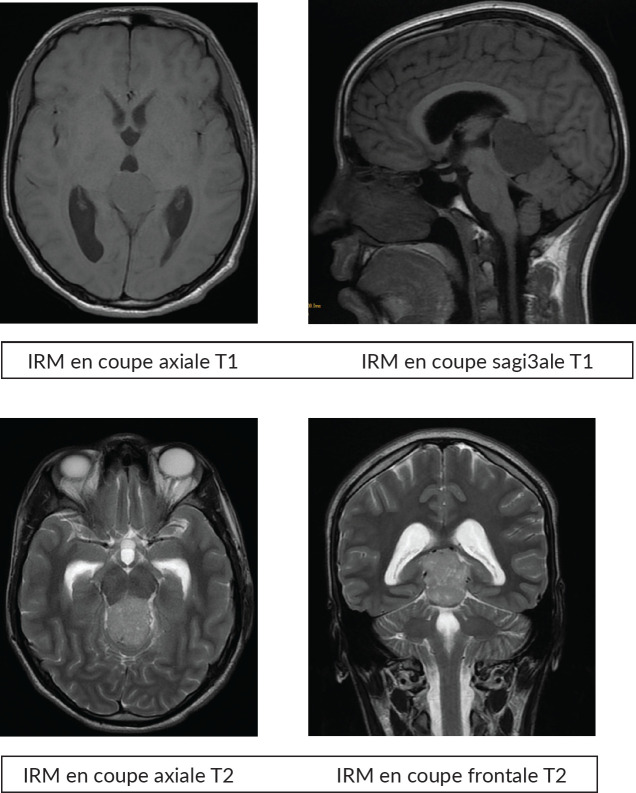
MRI images.

Brain magnetic resonance i maging (MRI) revealed an expansive lesion in the quadrigeminal cistern, measuring 25 × 37.5 × 39 mm in the pineal region. The mass had lobulated contours and was described as hypointense on T1-weighted imaging, with discrete hyperintensity on T2 and FLAIR sequences. It exhibited mild diffusion restriction and moderate enhancement after contrast administration. The lesion compressed the third ventricle, resulting in mild bi-ventricular obstructive hydrocephalus (Figure 1).

A stereotactic biopsy was performed to establish a definitive diagnosis. Given the tumour’s complex location in the pineal region – a deep intracranial zone surrounded by critical structures – the procedure was carried out under real-time CT guidance with a stereotactic frame ensuring millimetric precision. Following a thorough preoperative assessment and careful planning, a fine cannula was introduced via a cranial bore to obtain tumour samples. Pathological analysis revealed an undifferentiated neoplasm composed of small, round cells. Immunohistochemistry demonstrated strong positivity for placental alkaline phosphatase (PLAP), confirming the tumour’s germ cell origin, with a high Ki67 index of 40%, indicating moderate proliferative activity. The absence of GFAP, CK18, CD30, cytokeratin and alpha-fetoprotein (AFP) expression ruled out differential diagnoses such as gliomas and lymphomas. The procedure was well tolerated without major complications and played a crucial role in guiding subsequent therapy, which combined chemotherapy and radiotherapy.

The patient’s diplopia worsened, prompting a cerebral CT scan that confirmed active hydrocephalus. Consequently, he underwent ventriculoperitoneal shunting, leading to significant clinical and radiological improvement.

As part of the staging work-up, a spinal MRI was performed, which showed no evidence of spinal extension. Additionally, cerebrospinal fluid (CSF) cytological analysis was negative for tumour cells. Serum and CSF tumour marker assays for AFP and beta-human chorionic gonadotropin (β-HCG) were consistent with a non-metastatic, non-secreting seminoma.

Before initiating treatment, an endocrine evaluation was conducted to assess the thyrotropic and corticotropic axes, as well as the desmopressin pathway, all of which returned normal results. The patient also underwent sperm cryopreservation prior to therapy.

A multidisciplinary oncology board recommended neoadjuvant chemotherapy. He received three cycles of the bleomycin, etoposide and platinum regimen. The first cycle was well tolerated, with bleomycin administered on days 8 and 15, while etoposide and cisplatin were given daily from days 1 to 5. Renal function remained preserved, and pulmonary function tests revealed a mild restrictive ventilatory disorder.

The second cycle required dose adjustments due to moderate neutropenia (absolute neutrophil count: 60/mm^3^) and thrombocytopenia (70,000/mm^3^). These abnormalities led to a temporary postponement of bleomycin administration to day 15. Hemoglobin levels dropped slightly to 10 g/dL, but the patient tolerated treatment well under close monitoring. The third and final cycle was completed without major incidents. Post-treatment evaluations showed biological improvement, with hemoglobin at 12.5 g/dL, absolute neutrophil count at 3,280/mm^3^ and platelets at 398,000/mm^3^.

Post-chemotherapy assessment, including MRI, demonstrated significant tumour regression in the pineal region, although the lesion remained unresectable. Clinically, headaches and visual disturbances resolved completely. Treatment was well tolerated, with manageable side effects, including moderate fatigue, transient neutropenia and mild anemia, which resolved spontaneously. Following chemotherapy, the patient was referred for radiotherapy to consolidate treatment outcomes.

**Figure 2.**
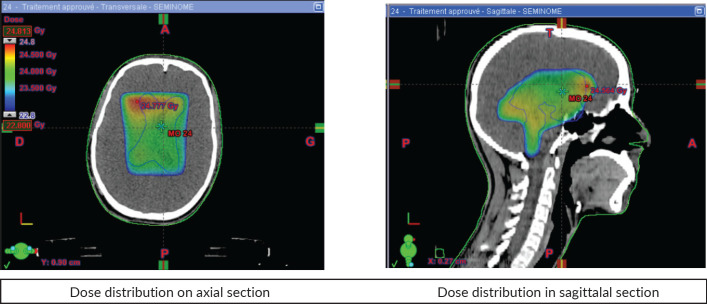
Dose distribution.

The patient received three-dimensional conformal radiotherapy, with a total dose of 24 Gy delivered in 12 fractions (2 Gy per fraction) to the tri-ventricular system. This was followed by a boost dose of 16 Gy in 8 fractions (2 Gy per fraction) targeting the pineal region, with good tolerance. The planning target volumes were generated with a 0.5 cm margin around the ventricles and the primary tumour, as visualised on MRI using fusion techniques (Figure 2). The maximum doses received by the brainstem and optic chiasm were 40 and 30 Gy, respectively.

Clinical and radiological follow-up was conducted every 3 months. At the 12-month follow-up, the patient remained in good control, with no signs of recurrence.

## Discussion

Intracranial GCTs represent less than 7% of all gonadal and extragonadal GCTs. They account for 0.5% to 3.2% of intracranial tumours in adults and children [3]. Germinoma tends to disseminate into the CSF and arachnoid and to give rise to neuraxial metastases. The other location of intracranial germinomas is the suprasellar, hypothalamic region. It is not unusual for germinoma to have both pineal and hypothalamic sites.

The histological appearance is often biphasic with diffuse membrane and cytoplasmic labelling for PLAP, a protein expressed by PGCs, but 5% to 10% of germinomas are PLAP negative [4].

Most of our current knowledge of germinal tumours (GTs) biology is based on molecular analysis of gonadal tumours, which are common and easily accessible. Intracranial GCTs, on the other hand, are much rarer and more complex to access, which limits the amount of tissue material available for genetic analysis and, therefore, explains the low number of molecular studies concerning them.

Intra cranial germinoma is included in the 5th edition of the World Health Organisation Classification of Tumours of the Central Nervous System published in 2021 [5]. Another classification of GTs into five types have recently been proposed, based on various characteristics including the nature of the cell of origin, histology, age, location, chromosomal abnormalities and the genetic imprinting status of the tumour tissue [6]. In this classification, only types I and II can develop intracranially. Type I GT are teratomas and pure vitelline tumours of the perinatal period and very young children, which rarely occur intracranially. Type II tumours are the most common and are divided into germinomatous and non-germinomatous tumours.

For intracranial localisation, the revealing symptomatology is often intracranial hypertension, damage to the cranial pairs or endocrine signs such as diabetes insipidus and pituitary insufficiency. There may be manifestations associated with spinal fluid dissemination, with metastases present in 10% to 30% of cases. They are usually latent and much more rarely cause spinal cord compression. Spinal metastases, particularly in malignant pineal region tumours, should be systematically investigated by CSF study and spinal imaging. Extraneurological metastases are much rarer [4].

MRI is the examination of choice for diagnosing these tumours, but if MRI is not available, a CT scan may be used in an emergency. Classically, tumours of the pineal region are iso-, hypo- or hyperintense in T1 with more or less homogeneous and limited contrast. There is a heterogeneous T2 hypersignal. Multi-planar slices can be used to determine extension to neighbouring structures, diffusion to subarachnoid spaces or dual localisation, which is essential for the therapeutic strategy: choice of approach, volume to be irradiated and response to chemotherapy, particularly in germinomas. Cerebral MRI must systematically be supplemented by spinal cord MRI, preferably performed preoperatively to look for spinal dissemination.

The discovery of a tumour in the pineal region should systematically lead to the measurement of a certain number of tumour markers in the blood, especially the CSF, which is the most sensitive. Sometimes, only one of the two areas may be positive. In principle, pure germinomas do not secrete b-hCG. However, 5% of them do secrete marginal amounts of b-hCG and would have a poorer prognosis [7]. Currently, many teams refer to non-secreting GCTs and secreting GCTs characterised by a level of AFP equal to or greater than 25 ng/dL and b-hCG equal to or greater than 50 IU/L in the blood and/or CSF [8].

Treatment of pineal region tumours is multidisciplinary and includes excisional surgery with ventricular bypass if there is hydrocephalus [9, 10]. Radiosurgery is reserved for tumours less than 3 cm in size, and the dose depends on the histological type. It achieves tumour control (total regression, partial regression and stabilisation) in 66% to 100% of cases, but does not prevent possible leptomeningeal spread [11, 12].

GCTs are highly chemosensitive; in pure germinomas, the role of platinum derivatives is essential and the chemotherapy protocol should use alternating VP16 (etoposide)/carboplatin and VP16/ifosfamide delivered every 3 weeks [4].

Because of their radiosensitivity, unresectable or non-operable germinomas can be treated with radiotherapy. Regardless of the technique used, precision in the delivery of the treatment is important, requiring the use of high-performance restraint masks to achieve precision of the order of 2 mm. The use of fusion techniques with MRI provides satisfactory accuracy in terms of delineating target volumes and organs at risk. Irradiation of the entire ventricular system is used in germinomas of the pineal region. A safety margin of 1 cm is recommended for the clinical target volume. The doses usually delivered range from 24 to 36 Gy.

In the case of a non-disseminated unifocal non-secreting pineal germinoma, irradiation will be limited to the ventricular system at a dose of 24 Gy. Ventricular radiotherapy after chemotherapy is probably a safe treatment to control subclinical disease [13, 14].

The main reason for limiting the extent of irradiation in young patients is to minimise potential adverse effects [15]. The use of chemotherapy for localised intracranial germinoma is justified by the reduction of sequelae by limiting irradiation of the brain and spine [16].

Proton therapy is playing an increasingly important role in the management of these tumours. Proton therapy provides better coverage of the target volume and spares more normal tissue than intensity-modulated radiation therapy for intra-renal GCTs in various locations [17, 18].

Our case shows that radiotherapy is a good alternative to surgery limiting postoperative adverse effects and providing excellent tumour local control.

## Conclusion

Seminomas of the pineal region remain a rare entity whose histological and radiological diagnosis is difficult. These tumours are highly radio-chemologically sensitive, and in the absence of surgery, radiotherapy represents an efficient alternative allowing local control and complication-free survival, especially with the new techniques available.

## Conflicts of interest

There is no conflicts of interest.

## Funding

There was no funding received.
